# An immune stratification reveals a subset of PD-1/LAG-3 double-positive triple-negative breast cancers

**DOI:** 10.1186/s13058-016-0783-4

**Published:** 2016-12-03

**Authors:** Giulia Bottai, Carlotta Raschioni, Agnese Losurdo, Luca Di Tommaso, Corrado Tinterri, Rosalba Torrisi, Jorge S. Reis-Filho, Massimo Roncalli, Christos Sotiriou, Armando Santoro, Alberto Mantovani, Sherene Loi, Libero Santarpia

**Affiliations:** 1 Oncology Experimental Therapeutics, IRCCS Clinical and Research Institute Humanitas, Via Manzoni 113, 20089 Rozzano-Milan, Italy; 2 Department of Oncology, IRCCS Clinical and Research Institute Humanitas, Rozzano-Milan, Italy; 3 Department of Pathology, IRCCS Clinical and Research Institute Humanitas, Rozzano-Milan, Italy; 4 Department of Surgery, IRCCS Clinical and Research Institute Humanitas, Rozzano-Milan, Italy; 5 Department of Pathology, Memorial Sloan Kettering Cancer Center, New York, NY USA; 6 Humanitas University, Rozzano-Milan, Italy; 7 Breast Cancer Translational Research Laboratory, Institut Jules Bordet, Université Libre de Bruxelles, Brussels, Belgium; 8 Department of Immunology and Inflammation, IRCCS Clinical and Research Institute Humanitas, Rozzano-Milan, Italy; 9 Division of Cancer Medicine and Research, Peter MacCallum Cancer Centre, East Melbourne, Victoria Australia

**Keywords:** Immune checkpoints, LAG-3, Prognosis, Triple-negative breast cancer, Tumor-infiltrating lymphocytes

## Abstract

**Background:**

Stromal tumor-infiltrating lymphocytes (TILs) are a robust prognostic factor in triple-negative breast cancer (TNBC). However, the clinical significance of TILs may be influenced by the complex landscape of the tumor immune microenvironment. In this study, we aimed to evaluate the composition and the functionality of lymphocytic infiltration and checkpoint receptors in TNBC.

**Methods:**

Formalin-fixed, paraffin-embedded tissues were retrospectively collected from a cohort of patients with early-stage TNBC treated with adjuvant anthracycline-based chemotherapy (n = 259). Results were validated in an independent cohort of patients with TNBC (n = 104). Stromal TILs were evaluated on hematoxylin-and-eosin-stained sections. The density of CD4+, CD8+, and FOXP3+ lymphocytes, and the expression of the immune checkpoints PD-1 and LAG-3, were assessed by immunohistochemical analysis.

**Results:**

The presence of elevated TILs positively correlated with the density of all T cell subtypes, especially cytotoxic CD8+ lymphocytes. We showed that increasing stromal TILs assessed as a continuous variable is an independent prognostic marker of prolonged relapse-free survival and overall survival in TNBC. Among immune subpopulations, CD8+ lymphocytes are the main effectors of anti-tumor immune responses. In two independent cohorts, we found that PD-1 and LAG-3 were concurrently expressed in approximately 15% of patients with TNBC. The expression of both checkpoint receptors positively correlated with the presence of TILs, but was not significantly associated with patient outcome.

**Conclusions:**

Overall, our data indicate that the evaluation of stromal TILs remains the most reliable immune prognostic marker in TNBC, and support the clinical evaluation of anti-PD-1/PD-L1 and anti-LAG-3 in a subset of patients with TNBC who have concurrent expression of both checkpoint receptors.

**Electronic supplementary material:**

The online version of this article (doi:10.1186/s13058-016-0783-4) contains supplementary material, which is available to authorized users.

## Background

Triple-negative breast cancer (TNBC) is usually characterized by an aggressive phenotype, associated with an increased risk of early recurrence within 3 years after diagnosis, and poor prognosis [1]. Current treatment approaches are limited to cytotoxic chemotherapy due to the lack of specific therapeutic targets [1]. Therefore, the identification of reliable prognostic markers and novel therapeutic targets may allow a better stratification of patients with TNBC, and provide the rationale for investigating innovative treatment strategies.

Recent evidence indicates that the immune microenvironment plays a key role in cancer progression and response to therapies [2]. Notably, the presence of tumor-infiltrating lymphocytes (TILs) is emerging as an important predictor of outcome and response to chemotherapy in TNBC [3–7]. However, the composition of the tumor immune microenvironment is very heterogeneous, and the functional significance of specific immune cell subpopulations remains poorly understood. Indeed, cytotoxic CD8+ T cells have been shown to be an independent favorable prognostic factor, while studies on CD4+ T helper and forkhead box protein 3 (FOXP3) + T regulatory cells have shown conflicting results [8].

Furthermore, even though TILs are able to identify and eliminate malignant cells, tumors have developed multiple mechanisms to maintain an immunosuppressive microenvironment, including the upregulation of inhibitory receptors, such as programmed cell death 1 (PD-1) and lymphocyte activation gene 3 (LAG-3) [9]. Recent findings demonstrate that the expression of immune markers related to immunosuppression is enriched in triple-negative/basal-like breast cancer, and correlate with prognosis and response to chemotherapy, supporting the evaluation of immunotherapy in TNBC [6, 10–14]. Thus, a deeper understanding of the composition and the functionality of lymphocytic infiltration could be useful to predict patients’ outcome, and to select patients with TNBC who may benefit from the addition of immune checkpoint drugs to standard chemotherapeutic regimens. The aim of the present study was to evaluate the composition and the functionality of lymphocytic infiltration in early-stage TNBC.

## Methods

### Patient cohorts and tumor samples

Formalin-fixed, paraffin-embedded (FFPE) tissues were retrospectively collected from 259 patients who underwent surgery at Humanitas Clinical and Research Institute (Rozzano - Milan, Italy). An additional independent cohort of TNBC samples (n = 104) collected from Humanitas Institutes (Catania and Castellanza, Italy) was used. All patients had histologically confirmed invasive ductal TNBC, and were treated with adjuvant anthracycline-based chemotherapy. The study was approved by the ethical committee of the Humanitas Hospitals. The study was conducted according to the “reporting of tumor marker studies” (REMARK) guidelines [15]. Clinical characteristics of patients included in this study are presented in Additional file 1: Table S1.

### Pathologic evaluation of TILs, immunohistochemical analysis and scoring

Histopathologic analysis of stromal lymphocytic infiltration was performed on full-face hematoxylin and eosin (HE)-stained sections according to Salgado et al. [16]. Stromal TILs were defined as the percentage of tumor stroma containing infiltrating lymphocytes. Areas of adjacent normal breast, *in situ* carcinoma, necrosis or fibrosis were not included in the evaluation. TILs were reported in 10% increments [3, 5]. We defined the lymphocyte-predominant breast cancer (LPBC) as TNBC with ≥50% infiltration of either tumor stroma or tumor nest. A binary cutoff ≥20% was also used to assess its potential to identify low-risk patients with TNBC stratified by nodal status, as previously described [17].

FFPE sections (3 μm) from TNBC samples were deparaffinized with xylene, rehydrated with a graded ethanol series (100%, 95%, 70%) to distilled water according to standard immunohistochemical protocols. Specificity of staining was determined by immunohistochemistry (IHC) on a set of cultured cell pellet blocks, normal specimens, and diverse tumor tissues in the form of whole sections, processed using the same fixative and processing methods as TNBC samples tested in the study [18–20]. The optimal concentration of each antibody was established performing serial titrations on serial FFPE sections. Antigen-retrieval conditions and detection methods were also optimized for each antibody to improve sensitivity and signal-to-noise ratio. Specificity was further determined by western blotting.

Reproducibility of antibodies was assessed with IHC analysis of serial FFPE sections stained under the same conditions on different days [20]. Briefly, heat-induced antigen retrieval was performed by placing slides in Tris-EDTA (pH9) or citrate (pH6) buffer for 20 minutes at 98 °C using a water bath. Tissue sections were cooled in buffer for 20 minutes before the treatment with Peroxidase Blocking Reagent (Dako) for 10 minutes. Slides were then incubated with Background Sniper (Biocare) for 20 minutes, and then with anti-CD4 (1:100, clone 4B12, Dako), anti-CD8 (1:100, clone C8/144B, Dako), anti-FOXP3 (1:100, clone 236A/E7, Abcam), anti-PD-1 (1:100, clone NAT105, Abcam), and anti-LAG-3 (1:200, clone 17B4, LS Bio) primary monoclonal antibodies. After washing in PBS, DAKO Envision systems (Dako) or MACH 1 Universal HRP Polymer (BioCare), and diaminobenzidine (DAB; BioCare) were used for chromogenic immunodetection, followed by counterstaining with hematoxylin. Negative control slides without primary antibody and positive controls for each marker were used for each immunostaining run. Full details on IHC protocols are provided in Additional file 1: Table S2.

IHC scoring was carried out as previously described [21, 22]. Briefly, each section was reviewed at low magnification. Positive lymphocytes in tumor stroma were counted in three high power fields (HPF; ×40; Olympus BX53), which represent the spectrum of staining seen on initial overview of the whole section, and displayed as average number of stained cells per HPF [21]. Patients were divided into two groups by the median value of CD4, CD8, and FOXP3 expression on TILs for statistical analyses. Patients with ≥5% of TILs expressing PD-1 or LAG-3 were considered positive [22].

Evaluation of TILs and IHC scoring were independently performed by two pathologists, who were blinded for patient characteristics and outcome. The mean value of two assessments was used for the current analyses. Agreement between the two pathologists was measured by calculating Cohen's kappa and the interclass correlation coefficients (ICCs). The inter-observer κ value for the categorical parameter LPBC was 0.63. The ICCs were 0.79 for TILs assessed as a continuous variable, 0.82 for CD4, 0.84 for CD8, 0.76 for FOXP3, 0.79 for PD-1, and 0.78 for LAG-3.

### Statistical analysis

Clinicopathological associations were tested using Fisher’s exact test and the Mann–Whitney *U* test for categorical and continuous data, respectively. Pearson correlation analysis was performed to evaluate the correlation between variables. Patients who developed tumor recurrence within 36 months after primary surgery were considered positive for tumor relapse, whereas patients who remained free of recurrence for the same time frame were defined as having non-relapsing tumors. Relapse-free survival (RFS) was defined as the time from surgery until the detection of distant recurrence. Overall survival (OS) was defined as the time from surgery to date of death. Patients who were alive (for OS) or recurrence-free (for RFS) were censored at date of last follow up. Survival analyses were performed by the Cox univariate proportional hazards model. For visualization purposes, Kaplan–Meier analyses were used for the survival curves test (Mantel-Cox log-rank test). Forest plots were used to visualize the results of Cox univariate analysis for RFS and OS. Multivariate Cox proportional hazard regression analysis was adjusted for relevant clinical covariates, including age at diagnosis, histologic grade, lymph node status, tumor size, and tumor stage. The likelihood ratio (LR) test was used to compare the different prognostic models. Changes in the LR values (ΔLRχ^2^) were used to quantitatively measure the relative amount of prognostic information of one model compared with another. All tests were two-sided and the level of statistical significance was set at *P* <0.05. Statistical analyses were performed using GraphPad Prism version 5, and StatsDirect version 3.

## Results

### Phenotypic profiling of TILs in TNBC

The majority of TNBC samples had lymphocytic infiltration in tumor stroma. Approximately 75% of patients with TNBC had at least 10% of stromal TILs (range 10–80%), while only 25% had virtual absence of lymphocytes (range 0–1%). The LPBC (TILs ≥50%) phenotype was found in 10.8% of patients with TNBC.

**Fig. 1:**
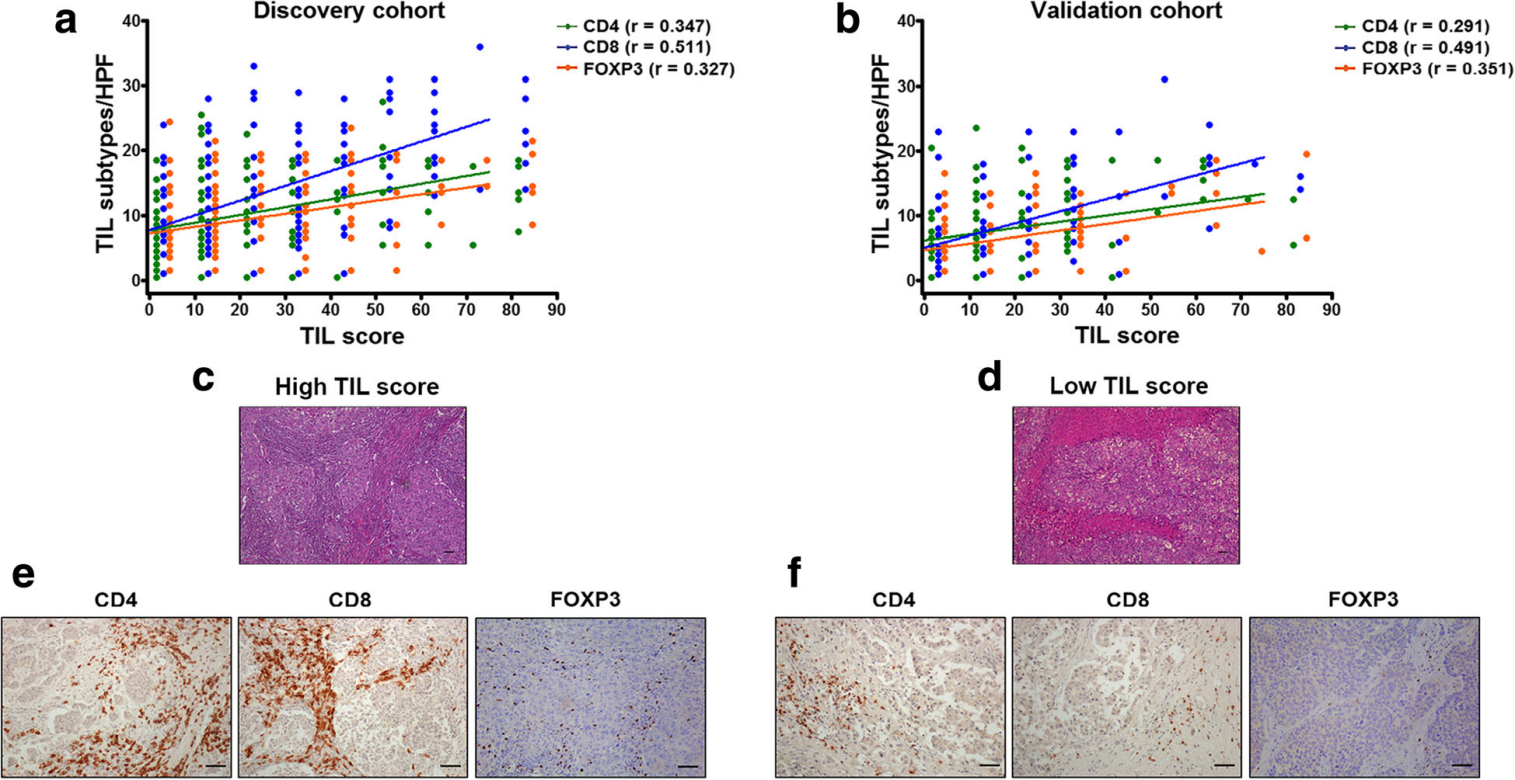
Distribution of tumor-infiltrating lymphocytes (*TILs*) and immune cell subpopulations in triple-negative breast cancer (*TNBC*). Immunophenotypic characterization of lymphocyte components showed that the presence of elevated TILs positively correlated with the density of CD4+, CD8+, and forkhead box protein 3 (*FOXP3+*) lymphocytes in TNBC in the discovery (**a**) and the validation (**b**) cohorts. Pearson’s correlation coefficients (*r*) for each cell subpopulation are shown. Cell density was scored by determining the average number of stained cells in three distinct high power fields (*HPF*). **c**-**f** Representative images of hematoxylin and eosin sections from TNBC samples with high (**c**) and low (**d**) TIL scores. Representative immunohistochemical staining of CD4, CD8, and FOXP3 in serial sections of TNBC specimens with high (**e**) and low (**f**) lymphocytic infiltration. *Scale bars* represent 50 μm

We further explored the nature of immune infiltrates by performing IHC of the main lymphocyte subsets. Immunophenotypic characterization of lymphocyte components showed that the presence of elevated TILs was positively associated with the density of CD4+ (*r* = 0.347) and FOXP3+ (*r* = 0.327) lymphocytes, and the strongest correlation was with CD8+ T cells (*r* = 0.511; Fig. 1a). These results were confirmed by analyzing an additional cohort of patients with TNBC (n = 104; Fig. 1b, Additional file 1: Table S1). Representative images of TNBC with different degrees of TILs and distinct lymphocyte subpopulations are depicted in Fig. 1c-f.

### Association of TILs with clinicopathological parameters and survival in TNBC

A lower stromal TILs content was associated with larger tumor size (*P* = 1.8E-02; Additional file 1: Table S3). There were no other significant associations between the variables examined and the presence of TILs, or with different immune cell subsets in a first TNBC cohort (n = 259; Additional file 1: Table S3). The data were confirmed in the validation cohort of TNBC (n = 104; Additional file 1: Table S4).

**Fig. 2:**
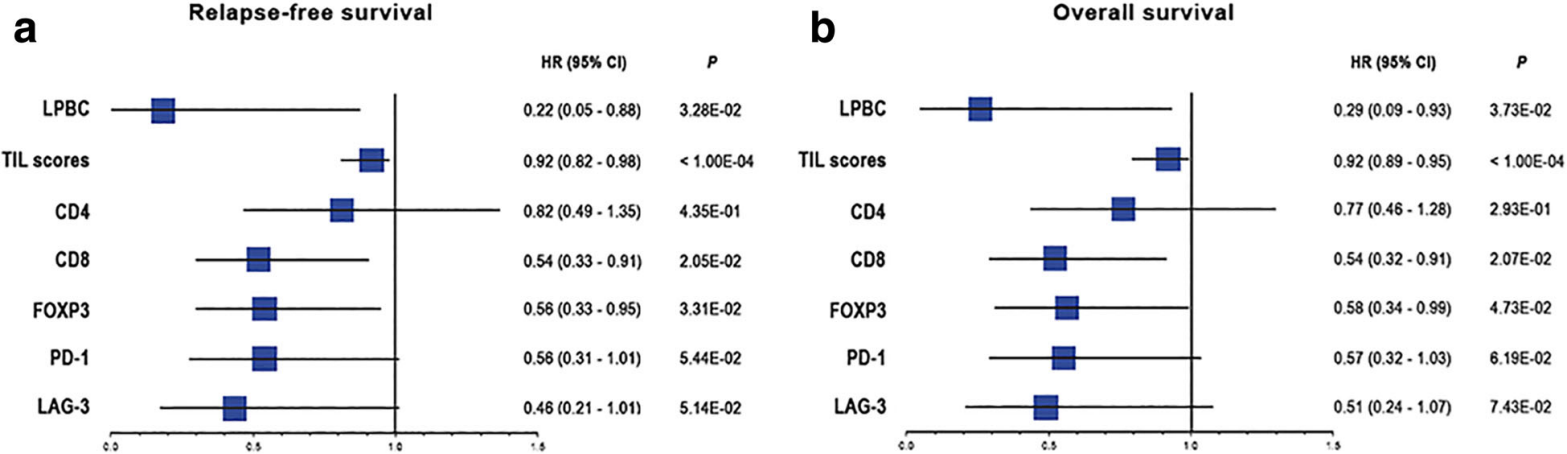
Univariate Cox regression analysis of tumor-infiltrating lymphocytes (*TILs*), immune markers and checkpoint receptors for relapse-free survival and overall survival in triple-negative breast cancer. Forest plot of hazard ratios (*HR*) with 95% confidence intervals (*CI*) for relapse-free survival (**a**) and overall survival (**b**), for lymphocyte-predominant breast cancer (*LPBC*) (cutoff value ≥50%), TIL scores (treated as a continuous variable for each 10% increment), immune markers (CD4, CD8, and forkhead box protein 3 (*FOXP3*); median values were used as the cutoff), and immune checkpoints (programmed cell death 1 (*PD-1*), and lymphocyte activation gene 3 (*LAG-3*); a cutoff value ≥5% was used)

**Fig. 3:**
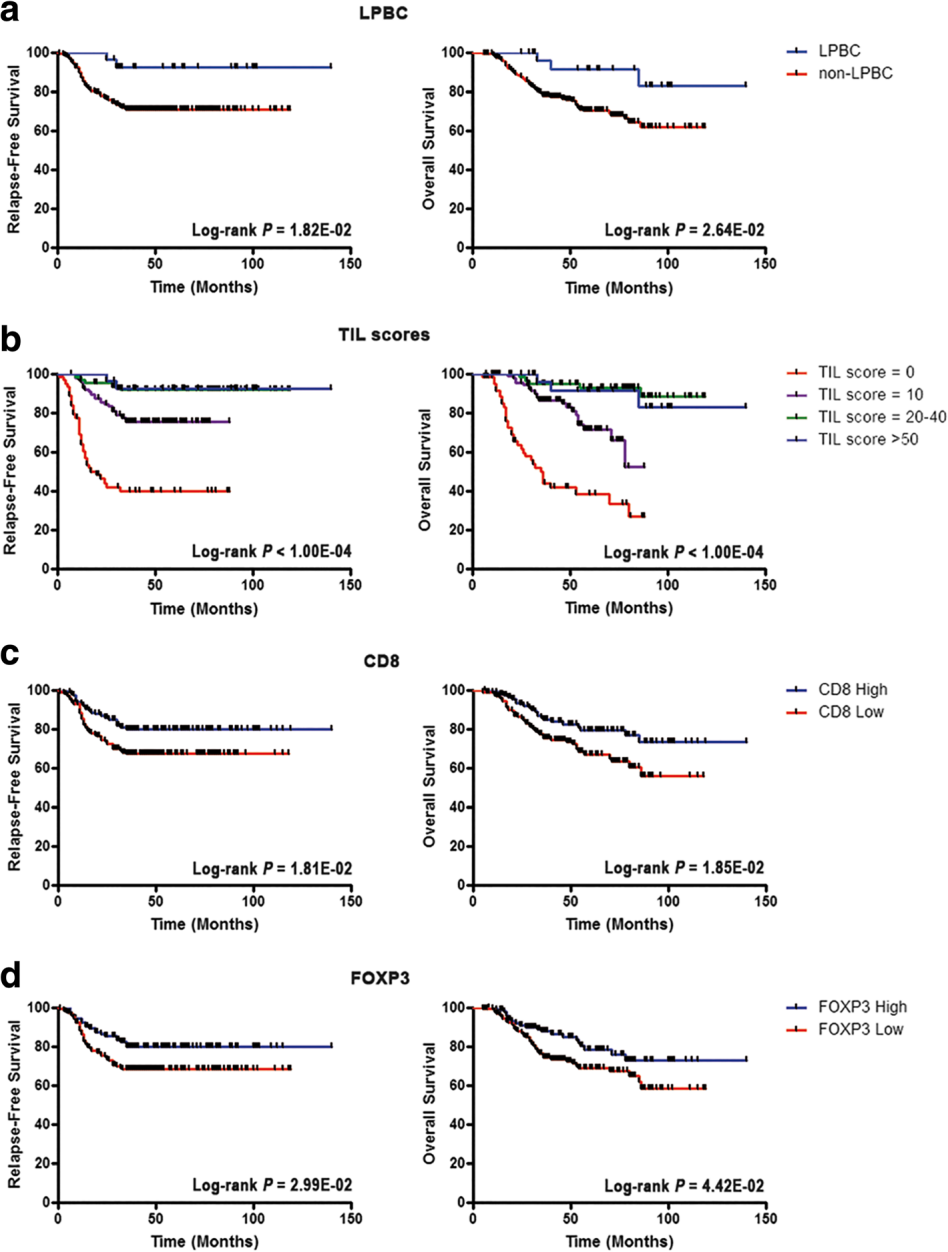
Prognostic value of stromal tumor-infiltrating lymphocytes (*TILs*) and immune cell subpopulations in triple-negative breast cancer. Kaplan–Meier curves of relapse-free survival and overall survival for binary lymphocyte-predominant breast cancer (*LPBC*) (cutoff value ≥50%) (**a**), continuous stromal TILs (grouped as 0 (range 0–1%) vs 10 (range 2–10%) vs 20–40 (range 11– 40%) vs 50–80 (range 41–80%)) (**b**), CD8 (median value) (**c**), and forkhead box protein 3 (*FOXP3*) (median value) (**d**). Curves were compared using the log-rank test

The association of LPBC, continuous TIL scores, and single immune components with RFS or OS in patients with TNBC was evaluated by Cox proportional hazard regression analyses (Fig. 2, Fig. 3, and Table 1). TILs assessed as a binary variable (LPBC vs non-LPBC) were associated with both RFS (hazard ratio (HR) = 0.22; 95% confidence interval (CI), 005 to 0.88; *P* = 3.28E-02) and OS (HR = 0.29; 95% CI, 0.09 to 0.93; *P* = 3.73E-02) in TNBC in univariate analysis (Fig. 2 and Fig. 3a), but had no prognostic value on multivariate analysis (Table 1). However, continuous TIL scores had a significant prognostic value for RFS (HR = 0.92; 95% CI, 0.82 to 0.98; *P* < 1.00E-04) and OS (HR = 0.92; 95% CI, 0.89 to 0.95; *P* < 1.00E-04) in TNBC (Fig. 2 and Fig. 3b). Cox multivariate analysis confirmed that TIL scores were independently associated with RFS (HR = 0.93; 95% CI, 0.89 to 0.96; *P* = 1.00E-04) and OS (HR = 0.93; 95% CI, 0.90 to 0.95; *P* = 1.00E-04) in TNBC (Table 1). Furthermore, continuous TIL scores added significant prognostic information for RFS (ΔLRχ^2^ = 31.35; *P* < 1.00E-04) and OS (ΔLRχ^2^ = 28.23; *P* < 1.00E-04) beyond that provided by standard clinicopathological variables (Table 2).

**Table 1:** Multivariate Cox regression analysis of tumor-infiltrating lymphocytes and immune markers for relapse-free survival and overall survival in triple-negative breast cancer

	Relapse-free survival			Overall survival		
Variable	HR	95% CI	*P* value	HR	95% CI	*P* value
LPBC	0.24	0.06–1.00	5.10E-02	0.32	0.10–1.03	5.60E-02
Age	1.09	0.66–1.80	7.28E-01	1.13	0.68–1.87	6.27E-01
Histologic grade	1.66	0.78–3.53	1.91E-01	1.85	0.87–3.94	1.09E-01
Nodal status	3.44	1.72–6.88	*5.00E-04*	3.19	1.59–6.39	*1.00E-03*
Tumor size	1.21	0.74–1.99	4.48E-01	1.27	0.77–2.09	3.48E-01
Tumor stage	1.34	0.76–2.35	3.12E-01	1.38	0.78–2.43	2.69E-01
TILs^a^	0.93	0.89–0.96	*1.00E-04*	0.93	0.90–0.95	*1.00E-04*
Age	0.97	0.58–1.61	8.97E-01	1.02	0.61–1.71	9.32E-01
Histologic grade	1.34	0.63–2.87	4.48E-01	1.76	0.83–3.75	1.40E-01
Nodal status	2.91	1.43–5.90	*3.10E-03*	2.59	1.27–5.28	*8.70E-03*
Tumor size	1.11	0.68–1.83	6.70E-01	1.15	0.70–1.90	5.73E-01
Tumor stage	1.46	0.82–2.62	2.00E-01	1.45	0.80–2.63	2.19E-01
CD8	0.58	0.34–0.97	*3.72E-02*	0.58	0.34–0.97	*3.88E-02*
Age	1.12	0.68–1.85	6.66E-01	1.16	0.70–1.93	5.58E-01
Histologic grade	1.71	0.80–3.65	1.67E-01	1.91	0.90–4.08	9.34E-02
Nodal status	3.46	1.73–6.90	*4.00E-04*	3.23	1.62–6.45	*9.00E-04*
Tumor size	1.20	0.73–1.97	4.80E-01	1.26	0.76–2.07	3.70E-01
Tumor stage	1.35	0.76–2.37	3.01E-01	1.35	0.76–2.40	2.99E-01
FOXP3	0.52	0.31–0.89	*1.71E-02*	0.55	0.32–0.94	*2.90E-02*
Age	1.10	0.67–1.83	6.98E-01	1.16	0.70–1.92	5.70E-01
Histologic grade	1.81	0.84–3.87	1.28E-01	2.01	0.94–4.30	7.27E-02
Nodal status	3.54	1.79–7.00	*3.00E-04*	3.30	1.67–6.53	*6.00E-04*
Tumor size	1.23	0.75–2.03	4.05 E-01	1.30	0.79–2.13	3.06E-01
Tumor stage	1.37	0.79–2.41	2.65E-01	1.38	0.78–2.42	2.66E-01

Multivariate analysis adjusted for age (≥50 vs <5 years), histologic grade (III vs – I-II), nodal status (1 vs 0), tumor size (>20 mm vs ≤20 mm), and tumor stage (III vs I–II). Significant *P* values are in italics. ^a^Treated as a continuous variable for each 10% increment. *CI* confidence interval, *HR* hazard ratio, *LPBC* lymphocyte-predominant breast cancer, *TILs* tumor-infiltrating lymphocytes

**Table 2:** Comparisons of added prognostic information

	Relapse-free survival		Overall survival	
Variable	ΔLRχ^2^	*P* value	ΔLRχ^2^	*P* value
CP + TIL score vs CP	31.35	*<1.00E-04*	28.23	*<1.00E-04*
CP + LPBC vs CP	5.20	*2.26E-02*	4.97	*2.58E-02*
CP + TIL score + CD8 vs CP + TIL score	0.43	5.12E-01	0.50	4.79E-01
CP + TIL score + FOXP3 vs CP + TIL score	1.02	3.12E-01	0.99	3.20E-01
CP + TIL score + CD8 + FOXP3 vs CP + TIL score	1.82	4.02E-01	1.70	4.27E-01

Significant *P* values are given in italics. *CP* clinicopathological variables (age, histologic grade, nodal status, tumor size, and tumor stage), *LPBC* lymphocyte-predominant breast cancer, *LR* likelihood ratio, *TILs* tumor-infiltrating lymphocytes, *FOXP3* forkhead box protein 3

Given that recent data suggest that a stromal TIL value ≥20% in early-stage TNBC could identify patients with good outcome across nodal categories, we performed Kaplan–Meier analysis to evaluate the prognostic value of this cutoff in TNBC stratified by nodal status (lymph node-negative and lymph node-positive) [17]. Overall, we found that patients with high levels of TILs (≥20%) had a better outcome compared with those with low TILs (<20%) in both nodal categories (Additional file 2: Figure S1).

Among lymphocyte subsets, the density of CD4+ cells was not significantly prognostic in TNBC (Fig. 2), while CD8+ lymphocytes were consistently associated with prolonged RFS and OS in both univariate (HR = 0.54; 95% CI, 0.33 to 0.91; *P* = 2.05E-02 for RFS; HR = 0.54; 95% CI, 0.32 to 0.91; *P* = 2.07E-02 for OS) and multivariate analysis (HR = 0.58; 95% CI, 0.34 to 0.97; *P* = 3.72E-02 for RFS; HR = 0.58; 95%CI, 0.34 to 0.97; *P* = 3.88E-02 for OS), indicating that cytotoxic CD8+ T lymphocytes are the main effectors of anti-tumor immune responses (Fig. 2, Fig. 3c, and Table 1). Furthermore, high FOXP3+ cells were also significantly associated with better survival in univariate (HR = 0.56; 95% CI, 0.33 to 0.95; *P* = 3.31E-02 for RFS; HR = 0.58; 95% CI, 0.34 to 0.99; *P* = 4.73E-02 for OS) and multivariate analysis (HR = 0.52; 95% CI, 0.31 to 0.89; *P* = 1.71E-02 for RFS; HR = 0.55; 95% CI, 0.32 to 0.94; *P* = 2.90E-02 for OS; Fig. 2, Fig. 3d, and Table 1). However, FOXP3+ cells were consistently associated with the density of CD8+ lymphocytes (*r* = 0.716), and the presence of FOXP3+ TILs was prognostically insignificant in TNBC stratified by the presence or absence of CD8+ cells (Additional file 1: Table S5).

Interestingly, we found that the infiltration of FOXP3+ cells tended to be associated with reduced survival in TNBC with low numbers of CD8+ lymphocytes (Additional file 1: Table S5), suggesting that the prognostic value of FOXP3+ cells was highly dependent on the concurrent presence of cytotoxic T lymphocytes. Of note, neither CD8+ nor FOXP3+ cells added consistent prognostic value for RFS (ΔLRχ^2^ = 0.43; *P* = 5.12E-01 for CD8; ΔLRχ^2^ = 1.02; *P* = 3.12E-01 for FOXP3) and OS (ΔLRχ^2^ = 0.50; *P* = 4.79E-01 for CD8; ΔLRχ^2^ = 0.99; *P* = 3.20E-01 for FOXP3) beyond that provided by the TIL score in the multivariate model (Table 2), suggesting that the evaluation of single immune components may not be as informative as the global evaluation of stromal TILs.

### Evaluation of the clinical relevance of immune checkpoints in TNBC

**Fig. 4:**
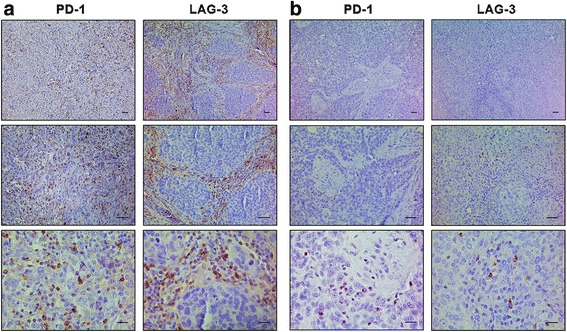
Programmed cell death 1 (*PD-1*) and lymphocyte activation gene 3 (*LAG-3*) protein expression in triple-negative breast cancer (TNBC). Representative immunohistochemical staining of PD-1 and LAG-3 in serial sections of TNBC samples with high (**a**) and low (**b**) lymphocytic infiltration. *Scale bars* represent 50 μm

To assess the functional status of TILs in TNBC, we analyzed the expression of the checkpoint receptors PD-1 and LAG-3 by IHC. PD-1+ and LAG-3+ TILs were present in approximately 30% and 18% of patients with TNBC, respectively (Fig. 4a, b). Concurrent expression of both immune checkpoints was observed in 15.4% of TNBC cases. We found that the expression of both PD-1 and LAG-3 positively correlated with the presence of TILs (*r* = 0.511; *r* = 0.576, respectively), particularly with CD8+ cells (*r* = 0.568; *r* = 0.490, respectively; Additional file 2: Figure S2a). By analyzing an additional cohort of patients with TNBC (n = 104; Additional file 1: Table S1), we confirmed that PD-1 and LAG-3 were concurrently expressed in 13.5% of patients, and that their expression was positively associated with TILs (*r* = 0.438; *r* = 0.537, respectively), and with CD8+ cells (*r* = 0.495; *r* = 0.467, respectively; Additional file 2: Figure S2b). Even though a trend for longer RFS was observed in univariate analysis, the presence of both PD-1+ and LAG-3+ TILs had no significant prognostic value in the discovery dataset (Fig. 2).

## Discussion

Recent evidence suggests that the presence of TILs is an important predictor of outcome and response to chemotherapy in TNBC [3–7]. In this dataset, we confirmed that increasing stromal TILs is an independent prognostic marker for prolonged RFS and OS in TNBC treated with adjuvant anthracycline-based chemotherapy. Studies evaluating the association between LPBC and survival have reported conflicting results [3–7]. Indeed, LPBC was not significantly associated with prognosis, likely due to the reduced number of events, and the small proportion of TNBC displaying this phenotype. Thus, further efforts are needed to improve the quantitative pathological assessment of TILs on HE-stained slides.

In agreement with recent data, we demonstrated that a value of ≥20% of stromal TILs could identify a group of low-risk patients with both lymph node-negative and lymph node-positive early-stage TNBC [17]. Furthermore, our findings suggest that patients with low numbers of TILs may benefit from the generation of an anti-tumor immune response, while boosting the lymphocyte activity (e.g. checkpoint inhibitors) might prove useful in patients with high numbers of TILs, associated with higher disease burden.

Although the presence of TILs reflects the activation of a local anti-tumor immune response, distinct immune cell subpopulations may have specific biological significance. In agreement with previous findings, we demonstrated that both CD8+ and FOXP3+ cells were associated with good outcome in patients with TNBC, and that the clinical significance of FOXP3+ lymphocytes was highly dependent on the concurrent presence of cytotoxic T cells [23–27]. Interestingly, when stratified based on the presence of CD8+ lymphocytes, a high infiltration of FOXP3+ cells trended towards reduced survival in TNBC patients with low numbers of CD8+ cells. These results suggest that CD8+ lymphocytes could be the main effectors of anti-tumor immune responses, and that the consistent correlation between FOXP3 positivity and cytotoxic lymphocytes may in part explain conflicting results reported in previous studies [8].

Overall, our findings indicate that the assessment of single immune components may not be as informative as the global evaluation of stromal TILs. However, the understanding of the biological role of different lymphocyte subpopulations warrants further investigations, and could be useful in selecting patients with TNBC who may benefit from the addition of specific immunomodulatory therapies to conventional chemotherapeutic regimens.

Even though TILs are emerging as important prognostic and predictive factors in TNBC, it is worth noting that many TNBC have few TILs, and even in the presence of massive lymphocytic infiltration, immunosuppressive mechanisms should be considered [28]. In this scenario, both radiotherapy and chemotherapeutic agents (e.g. anthracyclines) have been shown in preclinical models to be able to shape the tumor microenvironment, and to boost an effective immune response against tumor cells [29, 30]. These therapies could be rationally evaluated in combination with immunomodulatory drugs to synergize with pre-existing lymphocytes with tumoricidal activity, or to elicit a *de novo* local immune response in tumors lacking TILs.

Even though the immune system can recognize and eliminate malignant cells, tumors have developed multiple mechanisms to evade effective immunosurveillance, including the activation of the immune checkpoints PD-1 and LAG-3 [2, 9]. We demonstrated that PD-1+ and LAG-3+ TILs were present in approximately 30% and 18% of TNBCs, respectively, and that their presence in the tumor microenvironment tended to be associated with good prognosis in TNBC. The upregulation of these receptors, especially PD-1, has been classically described as a prominent immune resistance mechanism, and analyses performed on tissue microarrays have revealed an inverse correlation with outcome in patients with breast cancer [9, 31]. Indeed, double-positive PD-1/LAG-3 TILs have been recently demonstrated to show a more exhausted phenotype and functionality compared with single-positive or negative TILs in a preclinical model, likely leading to increased cancer immune evasion [32]. However, the role of co-inhibitory molecules in the modulation of the tumor immune microenvironment, and the mechanisms underlying T cell exhaustion and anergy are still poorly understood [9, 33]. Furthermore, it is worth noting that the activity of immune cells depends on the interaction with cancer cells, and recent findings support the idea that the functional relevance of checkpoint proteins is highly sensitive to the context (e.g. amount of antigen, topographical relationships with tumor cells and PD-L1-expressing cells) [9, 34]. Consequently, the evaluation of the clinical and biological significance of immune markers, especially those reflecting the activation status of lymphocytes, should be performed on whole tissue sections, reducing sampling bias due to tumor heterogeneity, and providing a more comprehensive understanding of the complex tumor-immune dynamics. Moreover, we found that the expression of both PD-1 and LAG-3 highly correlated with the presence of TILs, especially cytotoxic CD8+ cells.

Even though stratified analysis according to levels of lymphocytic infiltration was not performed due to the low number of cases in each subgroup, our results suggest that the presence of PD-1+ and LAG-3+ TILs in the tumor microenvironment may reflect the occurrence of an active, although partially exhausted, intratumoral immune response, rather than representing a global marker of immunosuppression. Accordingly, emerging evidence have revealed that local immunomodulatory factors (e.g. IFN-γ released by TILs), or the activation of oncogenic signaling pathways (e.g. the PI3K pathway) can promote the expression of PD-L1, which has been shown to be enriched in triple-negative/basal-like breast cancer, and to be associated with good outcome and response to chemotherapy in patients with TNBC [6, 10–14].

Interestingly, by analyzing two independent cohorts, we found that PD-1+ and LAG-3+ TILs were concurrently expressed in approximately 15% of TNBC cases. Thus, reversing the phenotype of exhausted T lymphocytes by targeting multiple inhibitory receptors may boost an effective anti-tumor immune response, and represent a novel valuable strategy to treat a subgroup of patients with TNBC. Recently, the blockade of the PD-1/PD-L1 pathway has shown promising clinical activity in patients with metastatic TNBC, although molecular preselection of the candidate patients for novel clinical trials would be valuable [33, 35–38]. Preclinical data demonstrates that anti-LAG-3 is mildly effective as monotherapy, but potently synergizes with anti-PD-1, suggesting that the combined immune checkpoint inhibition could enhance T cell activity and improve anti-tumor immunity [32]. Furthermore, the dual blockade of PD-1 and LAG-3 may exhibit less immune toxicity than that observed with the blockade of other immune receptors (e.g. CTLA-4).

Even though we confirmed the prognostic value of TILs in TNBC, the biological link between FOXP3+ and CD8+ lymphocytes, and the clinical relevance of checkpoint receptors in patients with TNBC with different levels of TILs, warrant further investigations. Despite these potential limitations, our findings support the clinical evaluation of combination immunotherapies with anti-PD-1/PD-L1 and anti-LAG-3 in a specific subset of patients with TNBC who have concurrent expression of both checkpoints.

## Conclusions

The presence of elevated TILs positively correlated with the density of all main T cell subtypes. The assessment of single immune components does not significantly improve risk stratification given by increasing stromal TILs, which remains an independent prognostic marker for TNBC treated with adjuvant chemotherapy containing anthracyclines. We have further demonstrated that the immune checkpoints PD-1 and LAG-3 are concurrently expressed in nearly 15% of tumors. The expression of PD-1 and LAG-3 is highly correlated with the presence of TILs, especially cytotoxic CD8+ cells, reflecting the occurrence of an effective intratumoral immune response. This study highlights the importance of different lymphocyte subpopulations for the selection of patients with primary TNBC who may benefit from immunomodulatory drugs. Our data support a clinical evaluation of anti-PD-1/PD-L1 and LAG-3 in combination with chemotherapy in a specific subset of patients with TNBC.

## Additional files

Additional file 1: Table S1. Patient characteristics. **Table S2.** Antibodies and immunostaining protocols. **Table S3.** Associations between tumor-infiltrating lymphocytes and clinicopathological features in triple-negative breast cancer (n = 259). **Table S4.** Associations between tumor-infiltrating lymphocytes and clinicopathological features in triple-negative breast cancer (validation cohort; n = 104). **Table S5.** Univariate Cox regression analysis of FOXP3 for relapse-free survival and overall survival in triple-negative breast cancer (n = 259) stratified by CD8+ tumor-infiltrating lymphocytes status. (PDF 90 kb)
Additional file 2: Figure S1. Prognostic value of the binary tumor-infiltrating lymphocytes (*TILs*) cutoff ≥ 20% in triple-negative breast cancer (*TNBC*) patients of the discovery cohort stratified by nodal status. **Figure S2.** Correlation between the expression of PD-1 and LAG-3 and the presence of CD8+ cells in triple-negative breast cancer (*TNBC*). (PDF 397 kb)

## Abbreviations

CI: confidence interval
CTLA-4: cytotoxic T-lymphocyte associated protein 4
FFPE: formalin-fixed, paraffin-embedded
FOXP3: forkhead box protein 3
HE: hematoxylin and eosin
HR: hazard ratio
IHC: immunohistochemistry
LAG-3: lymphocyte activation gene 3
LPBC: lymphocyte-predominant breast cancer
OS: overall survival
PBS: phosphate-buffered saline
PD-1: programmed cell death 1
PD-L1: programmed cell death ligand 1
RFS: relapse-free survival
TILs: tumor-infiltrating lymphocytes
TNBC: triple-negative breast cancer
