# Supplementary material for: An immune stratification reveals a subset of PD-1/LAG-3 double-positive triple-negative breast cancers
Source: Breast Cancer Res. 2016 Dec 3;18:121. doi: 10.1186/s13058-016-0783-4 (PMC5135782; doi:10.1186/s13058-016-0783-4)
Supplement: Additional file 1: Table S1. — Patient characteristics. Table S2. Antibodies and immunostaining protocols. Table S3. Associations between tumor-infiltrating lymphocytes and clinicopathological features in triple-negative breast cancer (n = 259). Table S4. Associations between tumor-infiltrating lymphocytes and clinicopathological features in triple-negative breast cancer (validation cohort; n = 104). Table S5. Univariate Cox regression analysis of FOXP3 for relapse-free survival and overall survival in triple-negative breast cancer (n = 259) stratified by CD8+ tumor-infiltrating lymphocytes status. (PDF 90 kb) [file 13058_2016_783_MOESM1_ESM.pdf]

Table S1 Patient characteristics

Clinical and pathological information	Discovery cohort	Validation cohort
**Patients (n)**	259	104
**Median age: years (IQR)**	50 (46 – 68)	53 (47 – 68)
**Median tumor size: mm (IQR)**	20 (14.5 – 35)	20 (15 – 45)
**T stage**		
T1	135 (52.1%)	52 (50.0%)
T2	94 (36.3%)	30 (28.8%)
T3	21 (8.1%)	17 (16.4%)
T4	9 (3.5%)	5 (4.8%)
**TNM Stage**		
I	78 (30.1%)	28 (26.9%)
II	99 (38.2%)	40 (38.5%)
III	82 (31.7%)	36 (34.6%)
**Node status**		
Negative	125 (48.3%)	53 (51.0%)
Positive	134 (51.7%)	51 (49.0%)
**Histological grade**		
grade 1	3 (1.2%)	1 (1.0%)
grade 2	52 (20.1%)	39 (37.5%)
grade 3	204 (78.7%)	64 (61.5%)
**Lymphovascular invasion**		
Absent	113 (43.6%)	-
Present	78 (30.1%)	-
Unknown	68 (26.3%)	104 (100%)
**Recurrence within 36 months**		
Yes	65 (25.1%)	14 (13.5%)
No	194 (74.9%)	90 (86.5%)
**TILs**		
Median (range)	10 (0 – 75)	10 (0 – 75)
IQR	2 – 25	1 – 25
**TIL scores**		
0	64 (24.7%)	27 (26.0%)
10	102 (39.4%)	28 (26.9%)
20	21 (8.1%)	14 (13.5%)
30	29 (11.1%)	19 (18.3%)
40	15 (5.8%)	6 (5.8%)
50	10 (3.9%)	2 (1.9%)
60	10 (3.9%)	5 (4.8%)
70	2 (0.8%)	1 (1.0%)
80	6 (2.3%)	2 (1.9%)
90	0 (0.0%)	0 (0.0%)
100	0 (0.0%)	0 (0.0%)

N number, *IQR* Interquartile range, *TILs* tumor-infiltrating lymphocytes

Table S2 Antibodies and immunostaining protocols.

Antibody	Type	Clone	Source	Dilution	Incubation	Positive controls	Antigen Retrieval	Detection system
CD8	Monoclonal	C8/144B	DAKO	1:100	30 min, RT	Human tonsil	Tris-EDTA, 20 min	EnVision FLEX, 30 min, RT
CD4	Monoclonal	4B12	DAKO	1:100	30 min, RT	Human tonsil	Tris-EDTA, 20 min	EnVision FLEX, 30 min, RT
FOXP3	Monoclonal	236A/E7	Abcam	1:200	1 h, RT	Lymph node	Tris-EDTA, 20 min	MACH1, 30 min, RT
PD-1	Monoclonal	NAT105	Abcam	1:100	1 h, RT	Lymph node	Tris-EDTA, 20 min	MACH1, 30 min, RT
LAG-3	Monoclonal	17B4	LS Bio	1:200	1 h, RT	Lymph node	Citrate, 20 min	MACH1, 30 min, RT

Table S3 Associations between tumor-infiltrating lymphocytes and clinicopathological features in triple-negative breast cancer (n = 259)

	Continuous TILs				CD4				CD8				FOXP3			
**Characteristic of patients**	**n**	**Median (range)**	**IQR**	***P***	**n**	**High (%)**	**Low (%)**	***P***	**n**	**High (%)**	**Low (%)**	***P***	**n**	**High (%)**	**Low (%)**	***P***
**Age at diagnosis**																
< 50	135	10 (0 - 75)	2 – 25	0.765	135	60 (44.4)	75 (55.6)	0.098	135	58 (43.0)	77 (57.0)	0.386	135	55 (40.7)	80 (59.3)	0.900
≥ 50	124	10 (0 - 75)	1 – 23.5		124	42 (33.9)	82 (66.1)		124	60 (48.4)	64 (51.6)		124	52 (46.0)	72 (54.0)	
**LN status**																
Negative	125	8 (0 - 72)	2 - 25	0.223	125	47 (37.6)	78 (62.4)	0.612	125	62 (49.6)	63 (50.4)	0.215	125	52 (41.6)	73 (58.4)	1.000
Positive	134	10 (0 - 75)	1 - 20		134	55 (41.0)	79 (59.0)		134	56 (41.8)	78 (58.2)		134	55 (41.0)	79 (59.0)	
**Histologic grade**																
G1-2	55	10 (0 - 72)	2 – 25	0.413	55	21 (38.2)	34 (61.8)	0.878	55	25 (45.5)	30 (54.5)	1.000	55	17 (30.9)	38 (69.1)	0.090
G3	204	10 (0 - 75)	0 – 23.5		204	81(39.7)	123 (60.3)		204	93 (45.6)	111 (54.4)		204	90 (44.1)	114 (55.9)	
**pTNM staging**																
I-II	177	10 (0 - 72)	2 – 25	0.338	177	74 (41.8)	103 (58.2)	0.275	177	85 (48.0)	92 (52.0)	0.284	177	76 (42.9)	101 (57.1)	0.498
III	82	10 (0 - 75)	1 – 20		82	28 (34.1)	54 (65.9)		82	33 (40.2)	49 (59.8)		82	31 (37.8)	51 (62.2)	
**Tumor size (mm)**																
≤ 20	138	10 (0 - 75)	2 – 25	*0.018*	138	55 (39.9)	83 (60.1)	0.899	138	71 (51.4)	67 (48.6)	0.060	138	58 (42.0)	80 (58.0)	0.800
> 20	120	8 (0 - 72)	1 – 13.5		120	46 (38.3)	74 (61.7)		120	47 (39.2)	73 (60.8)		120	48 (40.0)	72 (60.0)	
**LVI**																
Absent	113	8 (0 - 75)	2 – 23.5	0.860	113	47 (41.6)	66 (58.4)	1.000	113	60 (53.1)	53 (46.9)	0.056	113	58 (51.3)	55 (48.7)	0.186
Present	78	10 (0 - 72)	1 – 17.5		78	33 (42.3)	45 (57.7)		78	30 (38.5)	48 (61.5)		78	32 (41.0)	46 (59.0)	

Significant *P*-values are given in italics.
N number, *IQR* Interquartile range, *LN* lymph node, *LVI* lymphovascular invasion, *TILs* tumor-infiltrating lymphocytes, *FOXP3* forkhead box protein 3

Table S4 Associations between tumor-infiltrating lymphocytes and clinicopathological features in triple-negative breast cancer (validation cohort; n = 104)

	**Continuous TILs**				**CD4**				**CD8**				**FOXP3**			
**Characteristic of patients**	**n**	**Median (range)**	**IQR**	***P***	**n**	**High (%)**	**Low (%)**	***P***	**n**	**High (%)**	**Low (%)**	***P***	**n**	**High (%)**	**Low (%)**	***P***
**Age at diagnosis**																
< 50	51	12 (0 - 72)	1 – 30	0.507	51	16 (31.4)	35 (68.6)	0.266	51	16 (31.4)	35 (68.6)	0.831	51	8 (15.7)	43 (84.3)	0.458
≥ 50	53	10 (0 - 75)	2 – 23.5		53	11 (20.8)	42 (79.2)		53	15 (28.3)	38 (71.7)		53	12 (22.6)	41 (77.4)	
**LN status**																
Negative	53	10 (0 - 75)	1 – 25	0.860	53	15 (28.3)	38 (71.7)	0.657	53	20 (37.7)	33 (62.3)	0.088	53	10 (18.9)	43 (81.1)	1.000
Positive	51	10 (0 - 62)	2 – 25		51	12 (23.5)	39 (76.5)		51	11 (21.6)	40 (78.4)		51	10 (19.6)	41 (80.4)	
**Histologic grade**																
G1-2	40	10 (0 - 75)	1 – 21.7	0.288	40	13 (32.5)	27 (67.5)	0.256	40	14 (35.0)	26 (65.0)	0.385	40	8 (20.0)	32 (80.0)	1.000
G3	64	11 (0 - 62)	2 – 28.7		64	14 (21.9)	50 (78.1)		64	17 (26.6)	47 (73.4)		64	12 (18.8)	52 (81.2)	
**pTNM staging**																
I-II	68	10 (0 - 75)	1 – 28.7	0.978	68	18 (26.5)	50 (73.5)	1.000	68	22 (32.4)	46 (67.6)	0.504	68	12 (17.6)	56 (82.4)	0.607
III	36	10 (0 - 60)	2.7 – 25		36	9 (25.0)	27 (75.0)		36	9 (25.0)	27 (75.0)		36	8 (22.2)	28 (77.8)	
**Tumor size (mm)**																
≤ 20	54	13.5 (0 - 72)	1.7 – 30	*0.039*	54	18 (33.3)	36 (66.7)	0.116	54	20 (37.0)	34 (63.0)	0.133	54	14 (25.9)	40 (74.1)	0.085
> 20	50	5 (0 - 75)	1 – 20.2		50	9 (18.0)	41 (82.0)		50	11 (22.0)	39 (78.0)		50	6 (12.0)	44 (88.0)	

Significant *P*-values are given in italics.
N number, *IQR* Interquartile range, *LN* lymph node, *LVI* lymphovascular invasion, *TILs* tumor-infiltrating lymphocytes, *FOXP3* forkhead box protein 3
.

Table S5 Univariate Cox regression analysis of FOXP3 for relapse-free survival and overall survival in triple negative breast cancer (n = 259) stratified by CD8+ tumor-infiltrating lymphocytes status

	**TNBC with high CD8+ TILs**			**TNBC with low CD8+ TILs**		
**Variable**	**HR**	**95% CI**	***P* value**	**HR**	**95% CI**	***P* value**
			**Relapse-Free Survival**			
**FOXP3**	0.49	0.21 – 1.16	1.04E-01	1.04	0.47 – 2.35	9.14E-01
			**Overall Survival**			
**FOXP3**	0.49	0.21 – 1.15	1.03E-01	1.20	0.53 – 2.72	6.58E-01

HR hazard ratio, *CI* confidence interval, *FOXP3* forkhead box protein 3
