# Supplementary material for: An immune stratification reveals a subset of PD-1/LAG-3 double-positive triple-negative breast cancers
Source: Breast Cancer Res. 2016 Dec 3;18:121. doi: 10.1186/s13058-016-0783-4 (PMC5135782; doi:10.1186/s13058-016-0783-4)
Supplement: Additional file 2: Figure S1. — Prognostic value of the binary tumor-infiltrating lymphocytes (TILs) cutoff ≥ 20% in triple-negative breast cancer (TNBC) patients of the discovery cohort stratified by nodal status. Figure S2. Correlation between the expression of PD-1 and LAG-3 and the presence of CD8+ cells in triple-negative breast cancer (TNBC). (PDF 397 kb) [file 13058_2016_783_MOESM2_ESM.pdf]

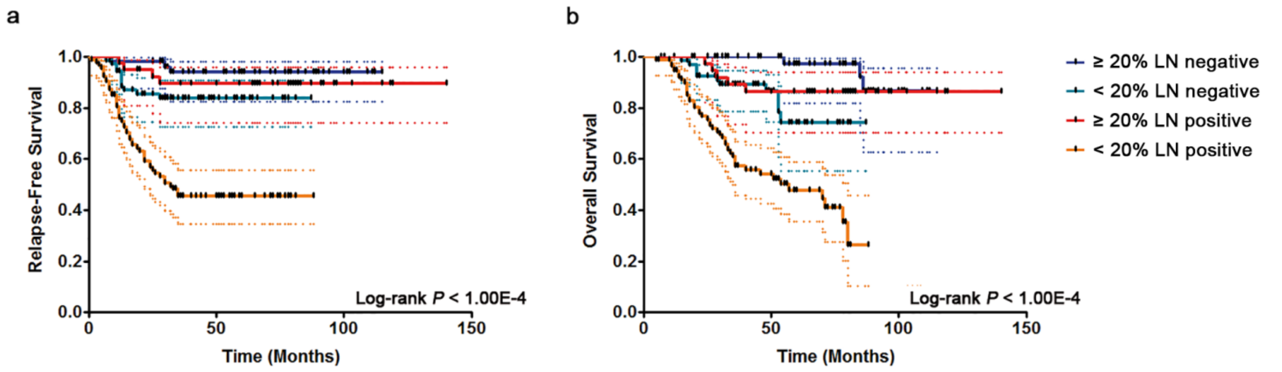

Fig. S1 Prognostic value of the binary tumor-infiltrating lymphocytes (*TILs*) cutoff ≥ 20% in triple-negative breast cancer (*TNBC*) patients of the discovery cohort stratified by nodal status. (**a**) Kaplan-Meier curves of relapse-free survival for binary 20% cutoff (≥ 20% vs < 20%) in TNBC patients stratified according to lymph node (*LN*) status (positive *v* negative) (*P* = 6.64E-02 in LN-negative TNBC patients; *P* < 1.00E-04 in LN-positive TNBC patients). (**b**) Kaplan-Meier curves of overall survival for binary 20% cutoff (≥ 20% vs < 20%) in TNBC patients stratified according to LN status (positive *v* negative) (*P* = 2.00E-03 in LN-negative TNBC patients; *P* < 1.00E-04 in LN-positive TNBC patients). Curves were compared using log-rank test. The dashed lines represent the 95% confidence intervals.

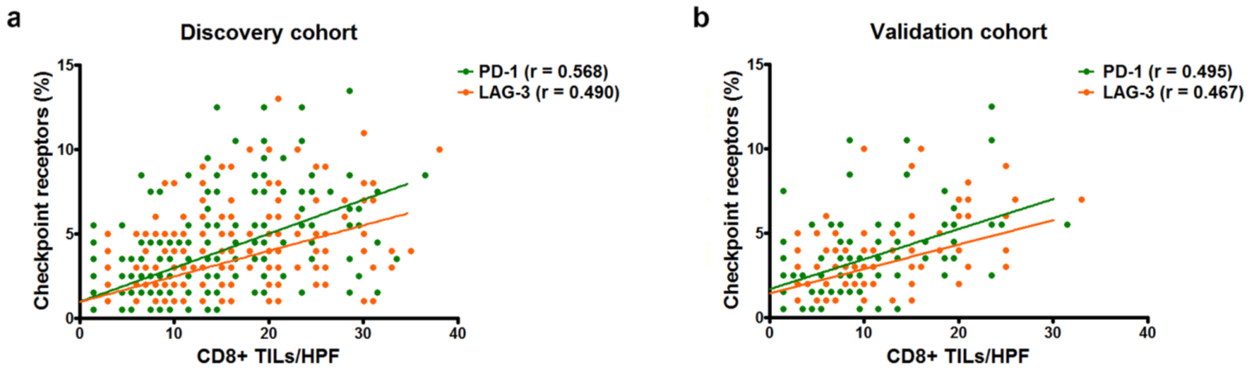

Fig. S2 Correlation between the expression of PD-1 and LAG-3 and the presence of CD8+ cells in triple-negative breast cancer (*TNBC*). The density of CD8+ T lymphocytes positively correlated with the expression of the checkpoint receptors PD-1 and LAG-3 in TNBC of the discovery (**a**) and the validation (**b**) cohorts. Pearson's correlation coefficients (*r*) for each cells subpopulation were shown. Cell density was scored by determining the average number of stained cells in 3 distinct high power fields (*HPF*).
